# Total cholesterol and low-density lipoprotein alterations in children and adolescents from Brazil: a prevalence meta-analysis

**DOI:** 10.20945/2359-3997000000508

**Published:** 2022-08-04

**Authors:** Thales Philipe Rodrigues da Silva, Larissa Loures Mendes, Virgínia Maria Jorge Barreto, Fernanda Penido Matozinhos, Camila Kümmel Duarte

**Affiliations:** 1 Universidade Federal de Minas Gerais Escola de Enfermagem Pós-graduação em Enfermagem Belo Horizonte MG Brasil Escola de Enfermagem, Programa de Pós-graduação em Enfermagem, Universidade Federal de Minas Gerais, Belo Horizonte, MG, Brasil; 2 Universidade Federal de Minas Gerais Escola de Enfermagem Departamento de Nutrição Belo Horizonte MG Brasil Departamento de Nutrição, Escola de Enfermagem, Universidade Federal de Minas Gerais, Belo Horizonte, MG, Brasil; 3 Universidade Federal de Minas Gerais Departamento de Enfermagem Materno-Infantil e Saúde Pública Belo Horizonte MG Brasil Departamento de Enfermagem Materno-Infantil e Saúde Pública, Universidade Federal de Minas Gerais, Belo Horizonte, MG, Brasil

**Keywords:** Adolescent, child, dyslipidemia, cholesterol, prevalence

## Abstract

**Objective:**

The aim of the present study was to evaluate the prevalence of total cholesterol (TC) and low-density lipoprotein (LDL) alterations in children and adolescents in Brazil.

**Materials and methods:**

A systematic review and meta-analysis of prevalence. The search for articles was carried out in the databases: Medline (PubMed), Embase, Scientific Electronic Library Online (SciELO), Latin American and Caribbean Literature in Health Sciences (Lilacs). The meta-analysis was performed using the random effects model. The I² test was used to identify heterogeneity.

**Results:**

The present metanalysis revealed a significant prevalence of altered lipid profile in children and adolescents in Brazil. Regarding lipoprotein fractions, the prevalence of altered TC level was 27.47% (95% CI 24.36-30.82), and a smaller prevalence was observed for LDL cholesterol (19.29% – 95% CI 15.21-24.16). The models revealed high heterogeneity (I² = 99%; p < 0.01), however the precise source of it was not identified; although type of school, age group, year and the region of Brazil appeared to influence the estimations of altered lipid profiles.

**Conclusion:**

An important prevalence of lipid alterations was observed among Brazilian children and adolescents. Those results reinforce the importance of knowing the lipid profile of children and adolescents to perform early interventions for this public.

## INTRODUCTION

Lipid profile alterations are characterized by quantitative alteration of a component of the serum lipids (increase in total cholesterol [TC], low-density lipoprotein [LDL] or triglycerides and decrease in high-density lipoprotein [HDL]) ( 1 ). International cutoff like the National Heart, Lung and Blood Institute (NHLBI) consider elevated TC, a cholesterol value > 200 mg/dL and a borderline value between 170 and 199 mg/dL; while the LDL value is pathological if it is > 130 mg/dL and borderline if > 110 mg/dL ( 2 ). However, in most Brazilian studies, the definition adopted is based on The Brazilian Society of Cardiology (BSC). The BSC classifies lipid profile for ages 2 to 19 years as follow: TC borderline 150-169 mg/dL; elevated ≥ 170 mg/dL and LDL borderline 100-129 mg/dL; elevated ≥ 130 mg/dL) ( 3 - 5 ).

Increases in LDL levels are the main predictor of CVD and LDL is the main component of TC ( 1 ). Therefore, both, TC and LD, are the focus of this study. In children and adolescents, lipid alterations can be risk factors to CVD; however, it usually occurs due to obesity ( 6 ). The association between lipid disorders and comorbidities, such as hypertension, obesity, and diabetes, are the main risk factors influencing the development of CVD ( 7 ). The Bogalusa Heart Study from the United States reported that atheromatous lesions in the aorta begin in childhood and it increases from 10 years of age until adulthood ( 8 ). The Bogalusa Heart Study revealed the presence of fatty streaks, which are precursors of atherosclerotic plaques, in the aorta and in the coronary bed of children and adolescents; those aortic injuries were correlated with elevated serum LDL levels ( 8 , 9 ).

Obesity, family medical history, physical inactivity, inadequate dietary patterns/habits are risk factors for lipid profile alterations in children and adolescents ( 10 , 11 ). Dietary habits and physical exercise are modifiable risk factors and, as such, can be subjected to intervention ( 12 , 13 ). However, to perform a better public health intervention it is important to understand the prevalence of lipid alteration in children and adolescents, as well as its geographical distribution, and other risk factors that may be related to the increase of the serum lipids.

Aiming to understand the impact of risk factors for CVD, the “ *Estudo de Riscos Cardiovasculares em Adolescentes* ” (ERICA) study ( 14 ) evaluated adolescents in public and private schools of Brazilian cities with populations >100,000. Results from that study, however, did not include children, and were limited to cities with large populations.

Several studies have reported the prevalence of TC and LDL alterations among children and adolescents in Brazil ( 15 - 18 ). However, the diversity of cutoff points adopted for the classification of altered TC and LDL among Brazilian studies makes it difficult to compare results internationally. Moreover, the prevalence of TC and LDL alterations and in children and adolescents from Brazil remains unclear. The aim of the present study was to evaluate the prevalence of TC and LDL alterations in children and adolescents in Brazil through meta-analysis and identify aspects that influence these rates.

## MATERIALS AND METHODS

The International Prospective Register of Systematic Reviews approved the research protocol (CRD42018103796). This systematic review followed the procedure suggested by the guidelines Preferred Reporting Items for Systematic Reviews and Meta-analysis (PRISMA) ( 19 ).

### Eligibility criteria

Eligibility criteria included cross-sectional and baseline of cohort studies investigating prevalence rates of altered lipid profile among children and adolescents in Brazil.

To be included, studies were required to describe the prevalence of TC and/or altered LDL levels of children (2-10 years old) or adolescents (>10-19 years old), and report information collected in a community or in schools of Brazil. The studies must have used the international cutoff criteria for diagnosis (NHLBI) or the latest cutoff proposed by the BSC ( 3 - 5 ). There were no restrictions towards publication date, language, or publication status. Studies that evaluated children or adolescents with specific health conditions (e.g., diabetes, psychological and/or genetic diseases, populations with specific congenital problems, genetic syndromes, endocrine or immunological dysfunction, or primary hypertension), were excluded. Interventional studies were also excluded due to its inclusion criteria and sample size estimations that usually do not allow to estimate prevalence adequately.

### Research strategy

Literature searches were performed in the Medline (PubMed), Embase, Scientific Electronic Library Online (SciELO), and *Literatura Latino-Americana e do Caribe em Ciências da Saúde* (Lilacs) databases using Medical Subject Headings (MeSH) terms and entries for PubMed and Embase, and DeCS (Health Sciences Descriptors) for the SciELO and Lilacs databases up to March 2022. Full-text versions of all potentially eligible articles were downloaded from the electronic databases or requested directly to the authors via e-mail. All searches were performed independently by two reviewers (VMJB and TPRS). Search strategies were tested using MESH and the related indexing terms for each database ( Table S1 ) with the keywords ‘‘dyslipidemia’’, “total cholesterol”, “low-density lipoprotein”, ‘‘prevalence’’, ‘‘children”, “adolescents” and ‘‘Brazil’’. No data and language restrictions were applied. An independent manual search of the reference lists of the retrieved articles was also performed.

### Study selection and data extraction

According to the eligibility criteria, two reviewers independently screened titles and abstracts and, later, read the full text articles. Disagreements were resolved by a third author. When studies with a sample already included in the review were identified, the study with the most complete data was considered. However, if the studies were identified from the same sample and, for example, one has a prevalence of TC and the other has a prevalence of LDL, both were included in the review. Observational studies were included if they provided cross-sectional data from baseline.

For data extraction, an electronic spreadsheet was created in which the following information was recorded: study name; authors; year of data collection; city; state; objective; age group; type and size of the sample; TC and LDL levels. TC and LDL levels were considered elevated according to the cutoff values determined by the authors in each study.

### Risk of bias within studies

Quality assessment of the studies was performed using the Newcastle-Ottawa Scale, with an adapted version of the scale for cross-sectional studies ( 20 ), with a maximum of 10 points for the least risk of bias study. Two authors (CKD and TPRS) evaluated the risk of bias. Although differences in quality assessment scores between investigators were unusual, they were resolved by consensus. The risk of publication bias across studies was explored with funnel plot asymmetry and Egger’s Test. Trim-to-fill correction was used in the presence of publication bias.

### Data analysis

The primary endpoints were the prevalence of altered TC and LDL cholesterol levels with the corresponding 95% confidence interval (CI). Summary measures were estimated for the total population and for subgroups defined according to age group, type of school, year of publication, and region of the country (i.e., Brazil). Brazil is geopolitically divided into five regions with at least three states on each region. Heterogeneity was assessed using the chi-squared test with statistical significance set at p < 0.10, and its magnitude was determined using the I² statistic. Meta-analysis was performed using a random effect model and weighted according to the inverse of variance. The meta-analyses were performed with articles using the same cutoff for diagnosis (NHLBI or BSC). Therefore, the same outcome has two forest plots: one with studies using BSC criteria and other for studies using the NHLBI criteria. Analyses were performed using the command “Metaprop” in RStudio version 3.4.4, adopting statistical significance at p < 0.05.

## RESULTS

**Figure 1:**
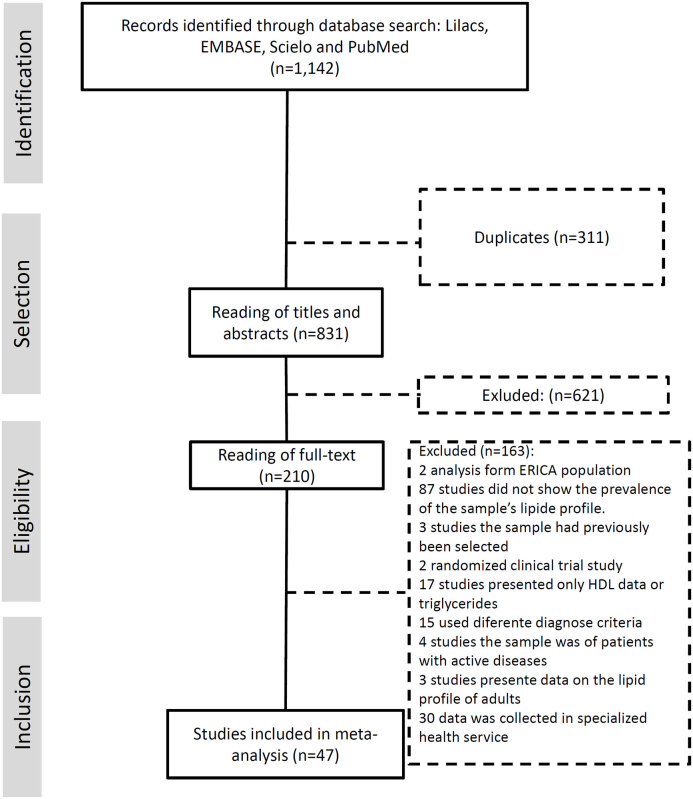
Systematic review and meta-analysis Flowchart.

In March 2022, the literature search identified 831 studies in the databases. After screening of titles and abstracts and, subsequently, full-text reading of the articles, 47 ( 14 , 21 - 66 ) studies were included in the present systematic review ( Figure 1 ). The characteristics of these studies are summarized in Table 1 . Sample sizes ranged from 95 to 38,069 across the studies. All regions of Brazil were represented; however, few studies from the Northern and Midwest states were found. Most studies were conducted in cities in the Southeast region, with emphasis on the states of São Paulo and Minas Gerais ( Table 1 ).

**Table 1:** Characteristics of the studies included in the prevalence meta-analysis

Study	Year	Sample		Age	Region	City	State		Population origin	Criteria		Quality assessment
										CT	LDL	
**CHILDREN**												
Almeida ( 21 )	2016	511	6 to 9 y		Southeast	Vitória	Espírito Santo		Public School	≥170	≥130	7
Barbalho ( 22 )	2017	150	6 to 10 y		Southeast	Lins	São Paulo		Public School	≥170	≥130	7
Filgueiras ( 23 )	2018	378	8 to 9 y		Southeast	Viçosa	Minas Gerais		Public and Private Schools	≥170	≥100	8
Nobre ( 24 )	2013	227	5 y		Southeast	Diamantina	Minas Gerais		–	≥170	≥130 ||	8
Rinaldi ( 25 )	2012	147	7,4 ± 1,4 y *		Southeast	Botucatu	São Paulo		not informed	≥200	≥130 ||	7
Silva ( 26 )	2013	677	6 to 10 y		Southeast	Santo André	São Paulo		Public School	≥200	≥130	6
Teixeira ( 27 )	2020	501	6 to 10 y		Southeast	Macaé	Rio de Janeiro		Public School	≥170	≥130 ||	8
**ADOLESCENTS**												
Arruda-Neta ( 28 )	2017	774	10 to 14 y		Northeast	João Pessoa	Paraíba		Public School	≥170	≥130 ||	7
Bauman ( 29 )	2020	635	10 and 16 y		Southeast	Montes Claros	Minas Gerais		Public School	≥170	≥130 ||	9
Beck ( 30 )	2011	660	14 to 19 y		South	Três de Maio	Santa Catarina		Public and Private Schools	≥170		8
Carvalho ( 31 )	2007	180	14 to 17 y		Northeast	Campina Grande	Paraíba		Public and Private Schools	≥170	≥110 ||	8
Chaves ( 32 )	2012	120	10 to 13 y		Southeast	Viçosa	Minas Gerais		Public School	≥170	≥130	9
Enes ( 33 )	2018	525	10 to 19 y		Southeast	Piracicaba	São Paulo		Public School	≥170	≥130 ||	5
Faria ( 34 )	2014	100	14 to 17 y		Southeast	Viçosa	Minas Gerais		Public School	≥170	≥130	8
Faria-Neto ( 14 )	2016	38,069	13 to 17 y		Brazil †				Public and Private Schools	≥170	≥130 ||	9
Gadelha ‡ ( 35 )	2019	236	15.1 ± 1.4 *		Northeast	Recife	Pernambuco		Cohort study of the public school	≥170	≥130	7
Gonçalves ( 36 )	2012	95	10 to 13 y		Southeast	Viçosa	Minas Gerais		Public School	≥170	≥130 ||	10
Guimarães ( 37 )	2019	997	12 to 18 y		South	Curitiba	Paraná		Public School	≥170	≥130 ||	8
Lunardi ( 38 )	2008	374	11.25 ± 0,28y *		South	Santa Maria	Rio Grande do Sul		Public and Private Schools	≥200	≥130 ||	9
Lunardi ( 39 )	2010	358	16 y		South	Santa Maria	Rio Grande do Sul		Public and Private Schools	≥200		9
Mastroeni ( 40 )	2016	222	15 to 17 y		South	Joinville	Santa Catarina		not informed	≥170	≥130	8
Melo ( 41 )	2016	196	11 to 19 y		Northeast	Natal	Rio Grande do Norte		Public School	≥170	≥130 ||	9
Pereira ( 42 )	2010	470	10 to 14 y		Northeast	Recife	Pernambuco		Public School	≥170	≥130 ||	9
Pinto ( 43 )	2011	117	14 to 17 y		Midwest	Brasília	Distrito-Federal		Public School	≥150	≥110 ||	8
Queiroz ( 44 )	2019	220	15 to 19 y		Northeast	João Pessoa	Paraíba		Public School	≥170	≥130 ||	9
Romero ( 45 )	2014	199	10 to 14 y		Southeast	Piracicaba	São Paulo		Public School	≥170	≥130 ||	9
Scheer ( 46 )	2019	394 §	13.3 ± 1,5y *		Southeast	Rio de Janeiro	Rio de Janeiro		Public School	≥170		7
Sousa ( 47 )	2013	250	11 to 18 y		Northeast	Salvador	Bahia		Public and Private Schools	>170	>110	8
Vasconcelos( 48 )	2008	140	12 to 16 y		South	São Mateus do Sul	Paraná		Public School	≥170	≥130 ||	9
**CHILDREN AND ADOLESCENTS**												
Alcântara-Neto ( 49 )	2012	937	7 to 14 y		Northeast	Salvador		Bahia	Public School	≥170		9
Bergmann ( 50 )	2011	1,294	7 to 12 y		South	Caxias do Sul		Rio Grande do Sul	Public and Private Schools	≥170		9
Burgos ( 51 )	2019	1,743	7 to 17 y		South	Santa Cruz do Sul		Rio Grande do Sul	Public and Private Schools	≥200	≥130 ||	9
Burgos ( 52 )	2015	1,254	7 to 17 y		South	Santa Cruz do Sul		Rio Grande do Sul	not informed	≥200	≥130 ||	8
Guimarães ( 53 )	2005	366	6 to 12 y		Northeast			Bahia	Public and Private Schools	≥170		6
Giuliano ( 54 )	2005	1,053	7 to 18 y		South	Florianópolis		Santa Catarina	Public and Private Schools	≥170		8
Gomes ( 55 )	2020	61,870	2 to 19y		Southeast	Campinas		São Paulo	Basic health unit	≥170	≥110	8
Moura ( 56 )	2000	1,600	7 to 14 y		Southeast	Campinas		São Paulo	Public School	≥170		10
Cunha ( 57 )	2014	399	6 to 15 y		South	Botuverá		Santa Catarina	Public School	≥170	≥130 ||	9
Pereira( 58 )	2009	494	2 to 19 y		Southeast	Itapetininga		São Paulo	Public School	≥170	≥110 ||	9
Quadros ( 59 )	2016	1,139	6 to 18 y		Northeast	Armagosa		Bahia	Public and Private Schools	≥170	≥130 ||	9
Quadros ( 60 )	2015	1,139	6 to 18 y		Northeast	Armagosa		Bahia	Public and Private Schools	≥170	≥130 ||	9
Reuter ( 61 )	2013	564	8 to 17 y		South	Santa Cruz do Sul		Rio Grande do Sul	Public and Private Schools	≥170	≥130	10
Reuter ( 62 )	2016	1,243	7 to 17 y		South	Santa Cruz do Sul		Rio Grande do Sul	Public School	≥200	≥130 ||	6
Ribas ( 63 )	2012	874	6 to 19 y		North	Belém		Pará	Public and Private Schools	≥170	≥130 ||	9
Ribas ( 64 )	2014	571	6 to 19 y		North	Belém		Pará	Public and Private Schools	≥170	≥110 ||	9
Ribas ( 65 )	2009	437	6 to 19 y		North	Belém		Pará	Private School	≥170	≥110 ||	8
Ribeiro ( 66 )	2010	3,106	6 to 18 y		South and Southeast	Belo Horizonte Florianópolis Blumenau		Minas Gerais e Santa Catarina	Public and Private Schools	≥200	≥130	9

Notes:

* Mean and standard deviation.

† ERICA – nationwide, school based and carried out in all regions of the country

‡ Data from 2012 and 2013

§ Data from regular schools.

|| Friedewald formula to estimate LDL.

Seven studies ( 21 - 27 ) exclusively evaluated lipid profile in 2,591 children, most of whom were from the Southeast region ( Table 1 ), ranging in age from 6 to 10 years. Twenty-two studies ( 14 , 28 - 48 ) examined exclusively adolescents (10 to 19 years old), covering 45,331 individuals, and were performed predominantly in the Northeast and Southeast regions of Brazil ( Table 1 ). In addition, 18 studies ( 49 - 66 ) evaluated simultaneously children and adolescents, totaling 24,400 individuals 2 to 19 years of age ( Table 1 ).

Some studies ( 14 , 21 - 23 , 26 - 51 , 53 , 54 , 56 - 66 ) evaluated children and adolescents from schools, and both public and private schools were included. Most studies followed the BSC criteria for diagnosing lipid profile alterations. Fewer studies ( 25 - 26 , 38 , 39 , 51 , 52 , 62 , 66 ) used the NHLBI diagnosis criteria for elevated TC and LDL. Some studies ( 21 - 24 , 26 - 29 , 32 - 37 , 40 - 42 , 44 , 45 , 48 , 57 , 59 - 61 , 63 ) used criteria from BSC but with the same cutoff for LDL values indicated by NHLBI (≥130 mg/dL).

Regarding the studies methodological quality evaluation, 44 studies were graded ≥ 7 of 10 stars. The articles were mostly downgraded due to lack of assessment of non-respondents ( Table 1 ).

A subgroup meta-analysis according to the cutoff criteria used to diagnose altered TC was performed. Pooled analysis for TC prevalence according to the NHLBI criteria indicated a tendency to a lower prevalence estimate (17.22% [95% CI 9.52-29.15]; I² = 99%) than the BSC criteria (27.47% [95% CI 24.36-30.82]; I² = 98%) (p-value between groups = 0.094) ( Table 2 and Figure S1A ). Therefore, the following analysis were performed for each diagnostic criterion, BSC and NHLBI.

**Table 2:** Summary of the prevalence rates according to the cutoff value used for total cholesterol and LDL cholesterol

Diagnostic criteria for the definition of the lipid alterations	Quoted studies	Number of subjects	Summary prevalence estimate	95% confidence interval	Forest plot	p-value
Total cholesterol						0.094
>170 mg/dL, BSC	35	117,159	27.47%	24.36-30.82	Figure S1A	
>200 mg/dL, NHLB	7	7,546	17.22%	9.52-29.15	Figure S1A	
Low-density lipoprotein						0.040
>110 mg/dL, BSC	7	64,139	19.29%	15.21-24.16	Figure S1B	
>130 mg/dL, NHLB	29	53,619	11.63%	7.45-17.71	Figure S1B	

Note: NHLBI: National Heart, Lung and Blood Institute; BSC: Brazilian Society of Cardiology.

A subgroup meta-analysis according to the region of Brazil revealed that the Southeast region had the highest prevalence (35.06% [95% CI 31.06-39.28]; I² = 99%) of elevated TC levels compared with the Northeast region (17.37% [95% CI 12.57-23.52]; I² = 95%) (p < 0.01) with the BSC criteria, with no differences when using NHLBI criteria ( Figures S2A and S2B ). A second subgroup meta-analysis examining the prevalence of elevated TC levels was performed according to the age group ( Figures 2 A e B ). Children exhibited a higher prevalence of altered TC levels (11.56% [95% CI 7.31-17.82]) than adolescents (4.78% [95% CI 3.45-6.59]) (p = 0.002) with the NHLBI criteria ( Figure 2A ), with no difference when using the BSC criteria ( Figure 2B ). A third subgroup analysis for altered TC was performed with the studies in which the samples came from school. The analysis were divided by type of school: private or public schools ( Figure 2C ). Children and adolescents in public schools presented a higher prevalence of altered TC levels (26.99% [95% CI 22.64-31.84]; I² = 96%) than those in private schools (18.15% [95% CI 12.78-25.11]; I² = 72%) (p = 0.034) ( Figure 2C ) with the BSC criteria, and no differences was observed when using NHLBI criteria. A fourth subgroup meta-analysis examining the year when studies were published revealed no difference (p = 0.391) in the prevalence of altered TC levels with the BSC criteria nor the NHLBI criteria ( Figures S2A and S2B ). To conclude the TC analysis, a subgroup metanalysis was performed by gender. No difference in the prevalence of altered TC level between girls and boys (p = 0.3439) was observed.

**Figure 2A:**
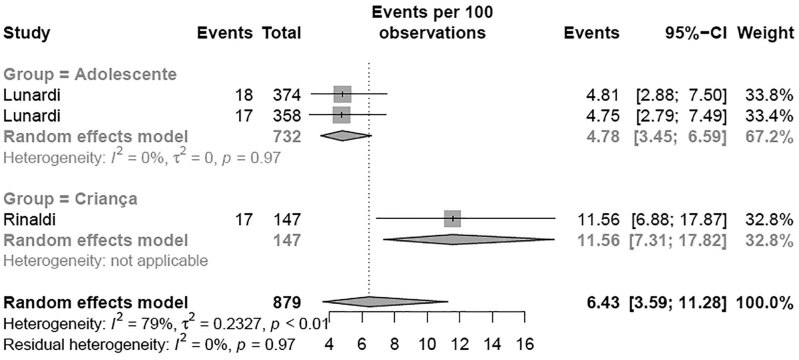
Prevalence of high total cholesterol for age group according NHLBI criteria. Note: p-value = 0.021.

**Figure 2B:**
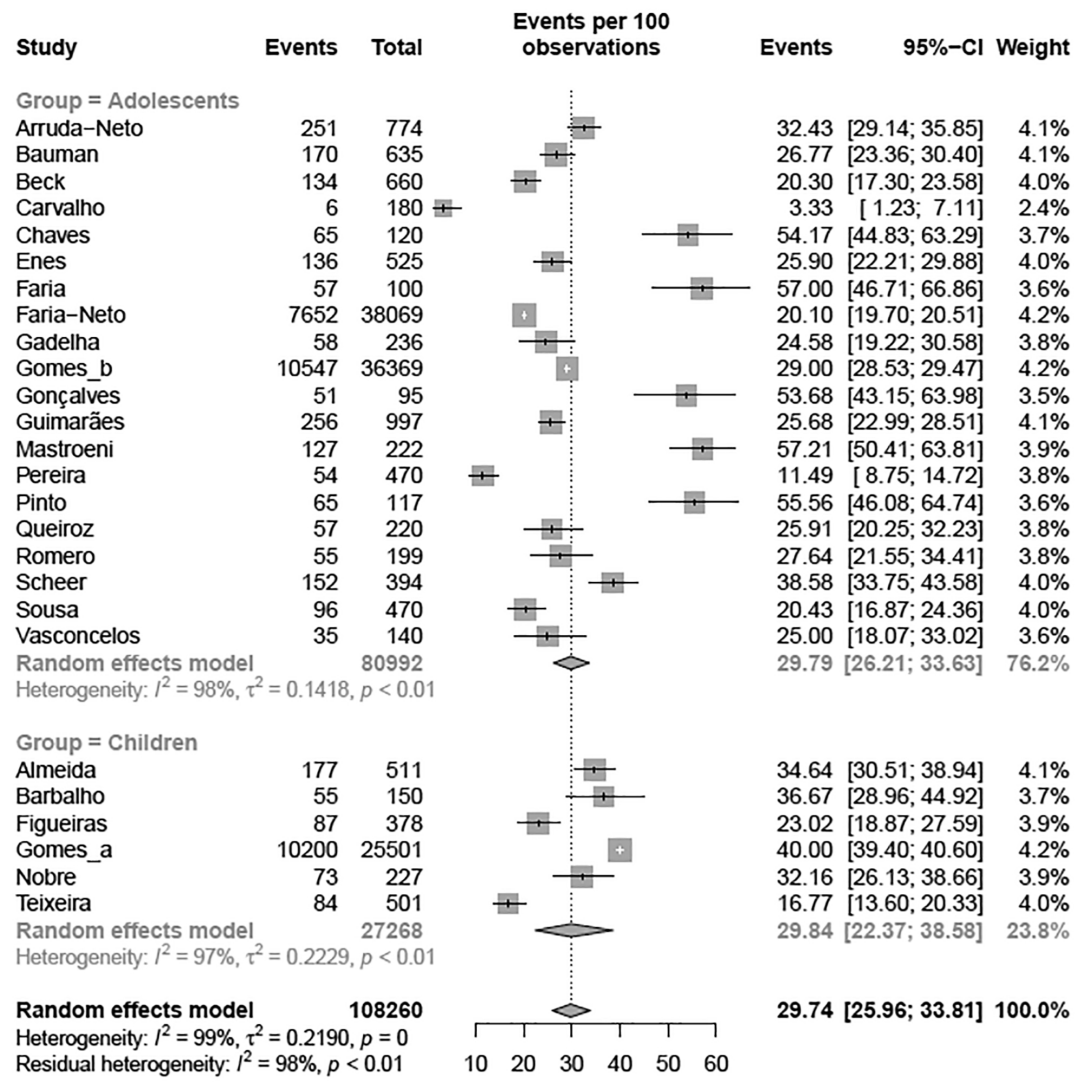
Prevalence of high total cholesterol for age group according BSC criteria Note: p-value = 0.990.

**Figure 2C:**
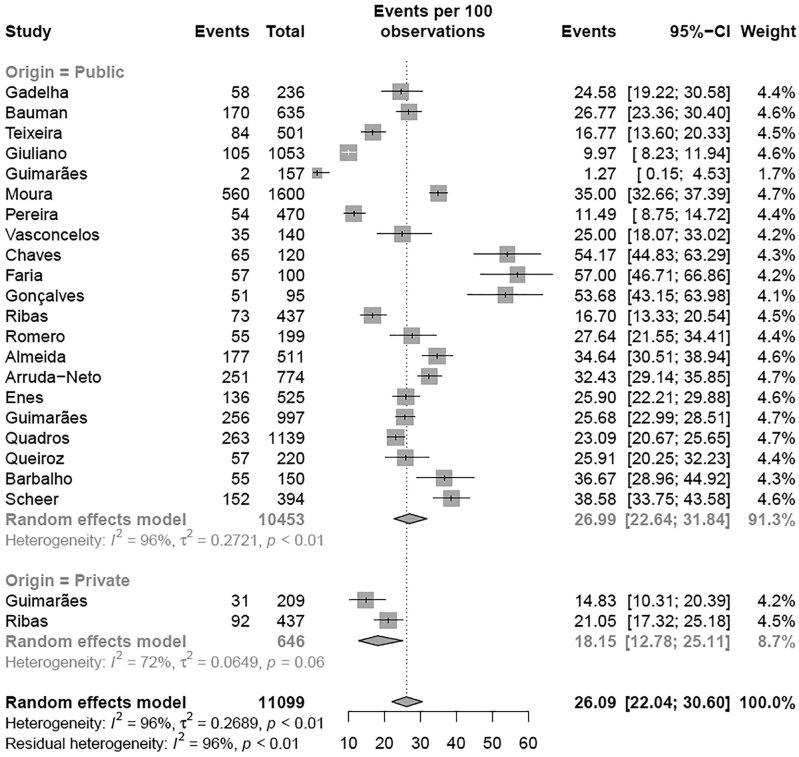
Prevalence of high total cholesterol for school administrative dependency according BSC criteria.

Note: p-value = 0.034.

Similar to TC, a subgroup meta-analysis according to the criteria adopted for altered LDL was performed. The estimated prevalence of elevated LDL levels in children and adolescents classified according to NHLBI (11.63% [95% CI 7.45-17.71]; I² = 99%) was different from the BSC criteria (19.29% [95% CI 15.21-24.16]; I² = 99%) (p-value between groups = 0.040) ( Table 2 and Figure S1B ) and the following analysis were performed for each criteria adopted. Regarding the regions of Brazil, the meta-analysis revealed that the South region had the highest prevalence of elevated LDL levels compared with the other regions (p < 0.001) using the NHLBI criteria ( Figure 3A ). When the regions of Brazil were analyzed with BSC criteria, the Southeast region had the highest prevalence of elevated LDL levels compared with other regions (p < 0.01) ( Figure 3B ). There was a tendency toward an elevated prevalence estimation of LDL in most recent studies (p < 0.05) with the NHLBI and BSC criteria ( Figures 4A and 4B ). There was no difference in the prevalence of altered LDL levels between girls and boys with the NHLBI criteria (p = 0.974), neither between age groups (children versus adolescents; p = 0.613) ( Figures S3A and S3B ). Subgroup analysis by gender were not performed with BSC criteria because only Ribas and Silva ( 63 ) provided data separated by gender and no difference between genders was observed in this study. Children exhibited a higher prevalence of altered LDL levels (35.00% [95% CI 34.42-35.59]) than adolescents (14.44% [95% CI 8.59-23.26]) (p < 0.002) with the BSC criteria ( Figure 5 ). No subgroup analysis was performed according to the type of school since all studies using BSC criteria were from public schools and all using NHLBI criteria were from private schools.

**Figure 3A:**
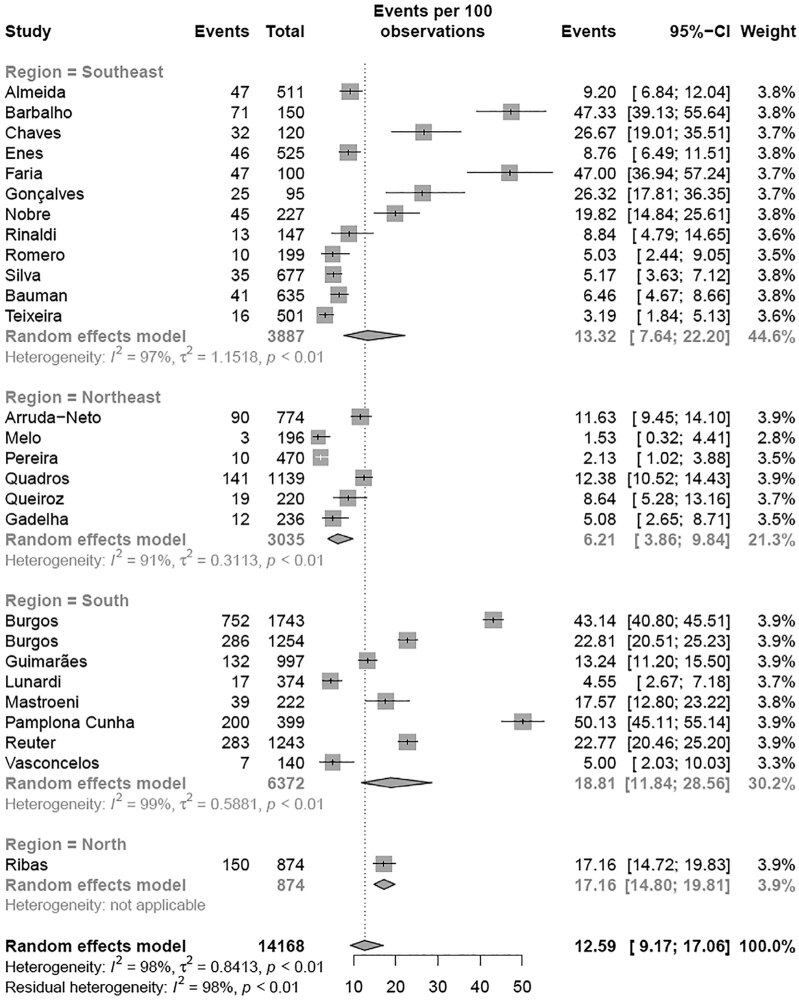
Prevalence of high LDL for region of Brazil according NHLBI criteria.

Note: p-value < 0.001.

**Figure 3B:**
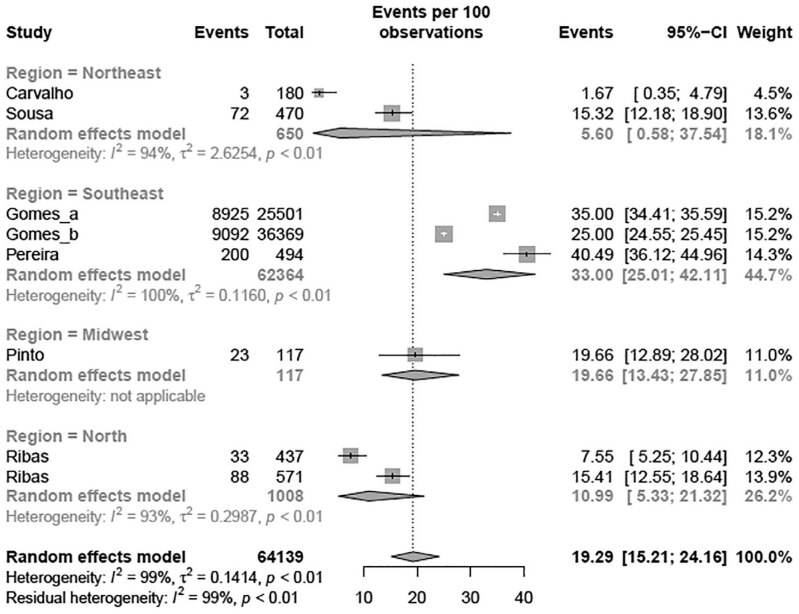
Prevalence of high LDL for region of Brazil according BSC criteria.

Note: p-value=0.003

**Figure 4A:**
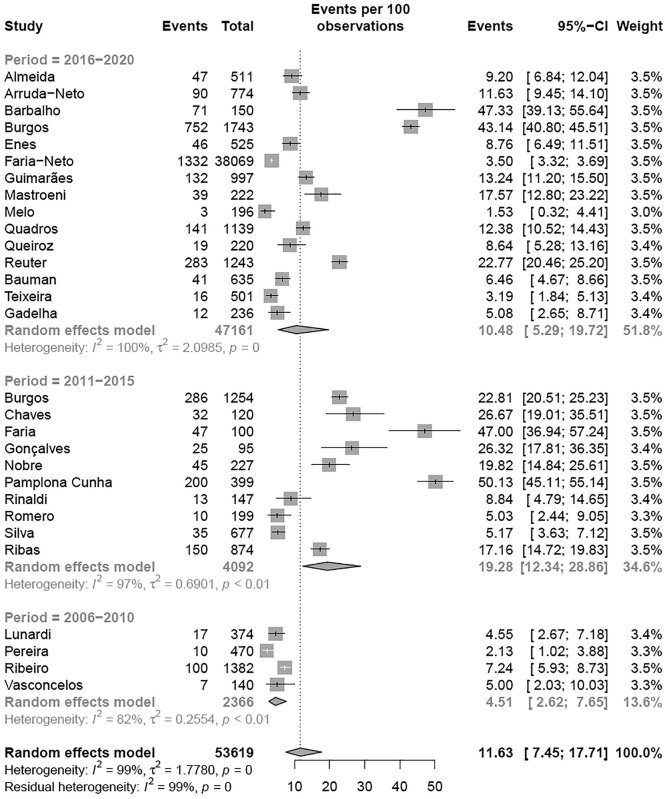
Prevalence of high LDL for period according NHLBI criteria.

Note: p-value < 0.001.

**Figure 4B:**
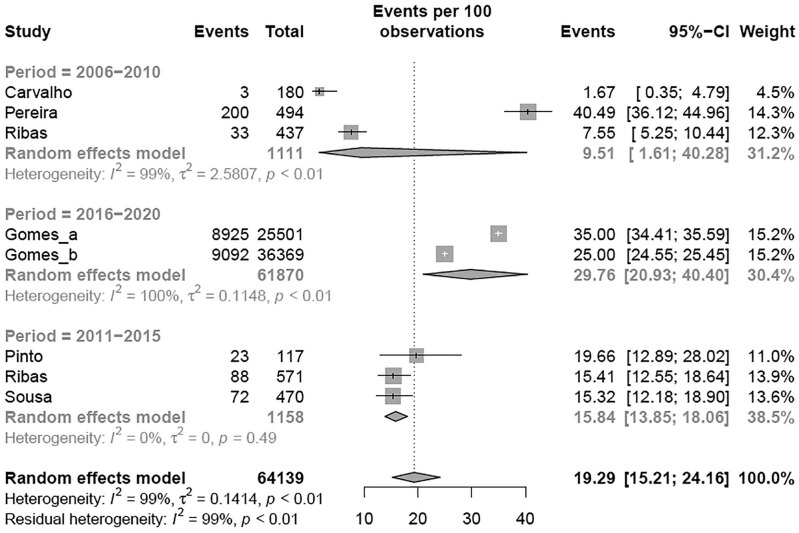
Prevalence of high LDL for period according BSC criteria.

Note: p-value<0.001

**Figure 5:**
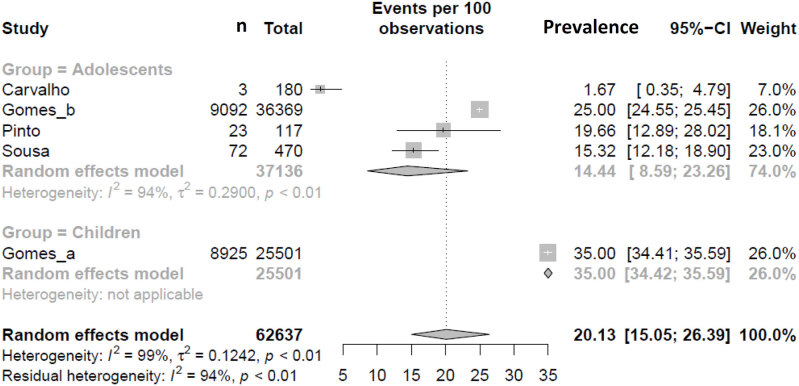
Prevalence of LDL for age group according BSC criteria.

Note: p-value< 0.001

The visual inspection of the funnel plot for TC and for LDL ( Figures S4A and S4B ) indicated publication bias. Therefore, the trim and fill correction was performed for TC and LDL ( Figures S4C , S4D , S4E and S4F ). The correction evidenced a very similar elevated TC prevalence estimation. On the other hand, the analysis for LDL evidenced that probably the prevalence of elevated LDL is underestimated in the previous analysis.

## DISCUSSION

The present meta-analysis revealed a significant prevalence of altered of TC and LDL alteration in children and adolescents in Brazil. Type of school, age group, year and the region of Brazil appeared to influence estimations of altered lipid profiles. In addition, the cutoff points used for the diagnosis of altered TC and LDL levels varied across the studies and influenced the prevalence estimation. We must recognize that the results on subgroup analysis were dependent of the diagnoses criteria adopted and the associations were tenuous. Although some associations were just seen when using one of the diagnoses criteria, a clinical tendency was seen on the same direction with the other criteria.

There was significant variability in the prevalence estimates for elevated TC and LDL levels among the studies. Some authors reported a very high prevalence, such as Faria and cols., who reported a rate of 57% in 100 adolescents for TC and 50% for LDL cholesterol ( 34 ), while other authors reported low values, 3.3% for altered TC and 1.7% for altered LDL in 180 adolescents ( 31 ). This discrepancies in prevalence estimation may be due to selection bias of small sample studies. However, sensitive analysis with studies with sample sizes greater than 300 subjects did not evidenced over or under estimations of lipid profile alterations prevalence (data not shown). The Brazilian Society of Pediatrics ( 67 ) recommends the use of the same cutoff values that does the BSC ( 3 - 5 ). However, international references ( 2 ) consider high a cholesterol value > 200 mg/dL and border line a value between 170 and 199 mg/dL, while the LDL value is pathological if > 130 mg/dL and border line if > 110 mg/dL. Burgos and cols. ( 51 ), who analyzed 1,743 children and adolescents in the South of Brazil and adopted the borderline criteria of the NHLBI (TC > 170 mg/dL and LDL > 110 mg/dL), reported prevalence of 60.75% and 43.14%, respectively ( 51 ). Although several differences were observed on the prevalence estimates in this meta-analysis, the prevalence of altered lipids in the Brazilian pediatric population was very high, even using the most conservative limits for the diagnosis, the NHLBI parameters.

Cutoff criteria is an especially important issue because it defines treatment strategies; therefore, validity studies of diagnostic tests are needed to identify the optimal cutoff points for TC and its fractions in the child and adolescent population. In 2011, in the city of Londrina, 1000 adolescents between 11 and 16 years old were subjected to lipid profile evaluation and classified according to 3 diagnostic criteria ( 68 ). Different prevalence estimates were found according to the distinct criteria: TC ( *BSC* 38.3%; National Cholesterol Education Program [NCEP] 11.2%; National Health and Nutrition Examination Survey [NHANES] 4.8%); and LDL (BSC/NCEP, 10.8% and NHANES, 5.9%). Overall, the prevalence of dyslipidemia according to each criterion was 61% (SBC), 28.6% (NCEP), and 24.2% (NHANES) ( 68 ).

Comparing the prevalence of elevated TC and LDL in public and private schools, the prevalence of altered TC and LDL in public schools were higher than in private schools. A Brazilian study about price and availability of food products with and without trans fatty acids in food stores near elementary schools located in low- and medium-income neighborhoods observed that cheaper products containing trans fats were more readily available than products without trans fats, promoting the consumption of less nutritious food by underprivileged children and adolescents ( 69 ). Reducing trans fatty acids intake by children may result in improvements on the lipid profile ( 70 ). Although some studies have shown a predominance of obesogenic environments with high ultra-processed food intake mainly in private schools ( 71 ), this type of food is consumed by all social classes ( 72 ) and in consequence by all children, from public to private schools. Our TC estimate in private schools came from just two studies, therefore we cannot assume that it happens the same in other private schools. Furthermore, in our meta-analysis, the Southeast and South, the richest regions of Brazil, exhibited higher rates of lipid profile alterations. Through systematic search, several studies were found, contributing to a representative sample size of the population of Brazilian children and adolescents. However, despite the large number of articles, the Northern and Central-Western regions were under-represented, which may have influenced the results according to region.

Another finding of the present study was the increase in the prevalence of elevated TC and LDL levels between 2000 and 2019. A tendency towards increase in prevalence rates was seen mainly after 2010. The pattern of food consumption in Brazilian population has been changing over the past few decades, with an increase in the consumption of ultra-processed foods ( 73 ). The early and abusive consumption of ultra-processed foods is one of the most important factors associated with the increased prevalence of obesity and the risk for metabolic complications ( 3 , 7 ), such as changes in lipid profile ( 4 , 5 , 67 , 74 - 78 ).

Another important factor in childhood and adolescence is the low level of physical activity and the increase of a sedentary lifestyle over the years ( 79 ). When it is associated with changes in eating habits, this behavior may contribute to increases in obesity and metabolic changes, including dyslipidemia ( 12 , 18 ). Data on Brazilian adolescents from all capitals of Brazil, indicates a prevalence of leisure-time physical inactivity around 54.3% ( 79 ). The Brazilian Society of Sports Medicine recommends regular physical activity and highlights the benefit of improved lipid profiles and believes there is an association between physical inactivity, obesity and dyslipidemia, and that obese children may become obese adults prone to illnesses related to weight gain ( 80 , 81 ). All this changes probably had some influence on the findings, although a selection bias could be present, especially in light of the profound heterogeneity reported on the analysis.

Higher rates of TC were detected in children when compared to adolescents, but no differences between boys and girls was observed. Lipoprotein concentrations changes considerably with normal growth and maturation, and varied according to sex ( 82 - 86 ). During pubertal growth, cholesterol is included in growing cells, leading to decreases in serum lipid values ( 87 ) but it increases on the later adolescence, approaching adult concentrations ( 84 ). Our sample of adolescents are mainly from individuals on the late phase (mean age 14.7 years old; data not shown). Therefore, screening recommendations should consider fluctuations in serum lipid levels during growth and sexual maturation. It is recommended that every child undergo a determination of TC level at 10 years of age by means of an examination of digital pulp capillary blood ( 4 ). Because adolescence is a critical period of life for the onset or persistence of obesity and its complications ( 86 ), knowing the nutritional status of this segment of the population is important.

### Strengths and limitations

Finally, it should be noted that the present meta-analysis was designed according to the standards recommended by the Cochrane Collaboration ( 87 ) and adhered to the Preferred Reporting Items for Systematic Reviews and Meta-Analyses: The PRISMA Statement ( 19 ) but it still presents some limitations. One of the main limitations of the present study was the high heterogeneity, the origin of which could not be explained. Although several subgroup analyses were performed to identify the source of heterogeneity, it remained elusive. Another limitation is the fact that information regarding nutritional status, dietary intake and physical activity was not reported in the articles and, consequently, was not analyzed in this study. This may also have contributed to the high heterogeneity. Most studies estimated the LDL with Friedewald’s formula, but no information was provided regarding patients with triglycerides over 400 mg/dL and if those patients were included it may have led to a biased result.

In conclusions, the present study indicates a high prevalence of altered – if not abnormal – lipid levels among children and adolescents in Brazil. These results reinforce the importance of knowing the lipid profile of children and adolescents to perform early interventions for treatment as well as to promote healthy habits that lead to prevention of lipid profile alterations and its consequences.

## Figures and Tables

**Figure S1A:**
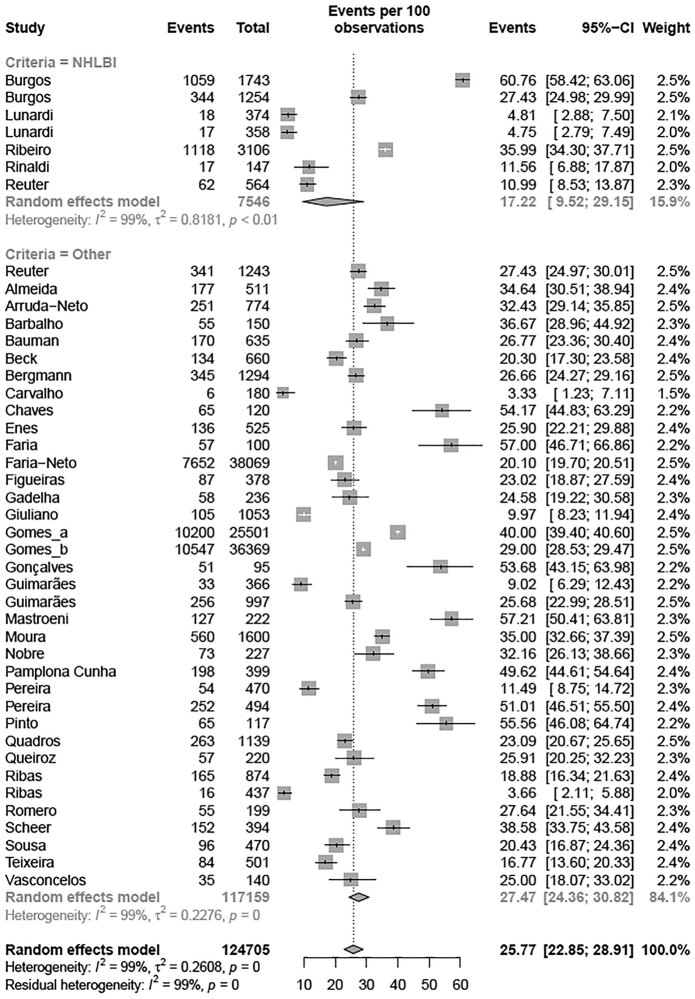
Prevalence of TC according to diagnose criteria.

Notes: p-value = 0.094.

**Figure S1B:**
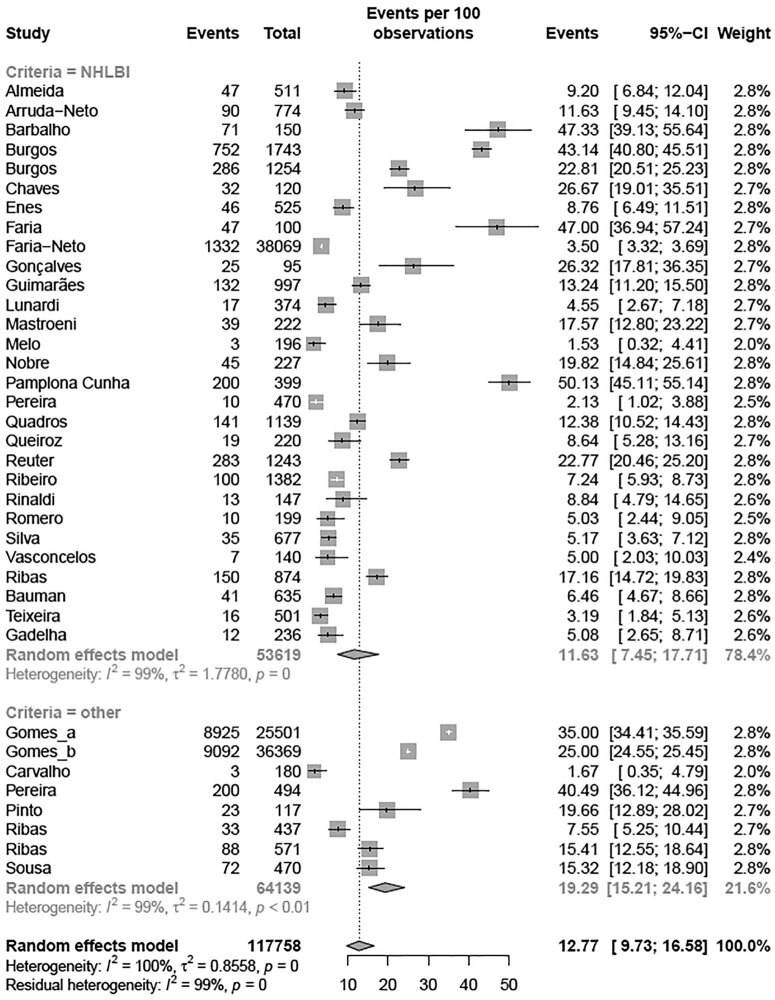
Prevalence of high LDL according to diagnose criteria.

Notes: p-value = 0.040

**Figure S2A:**
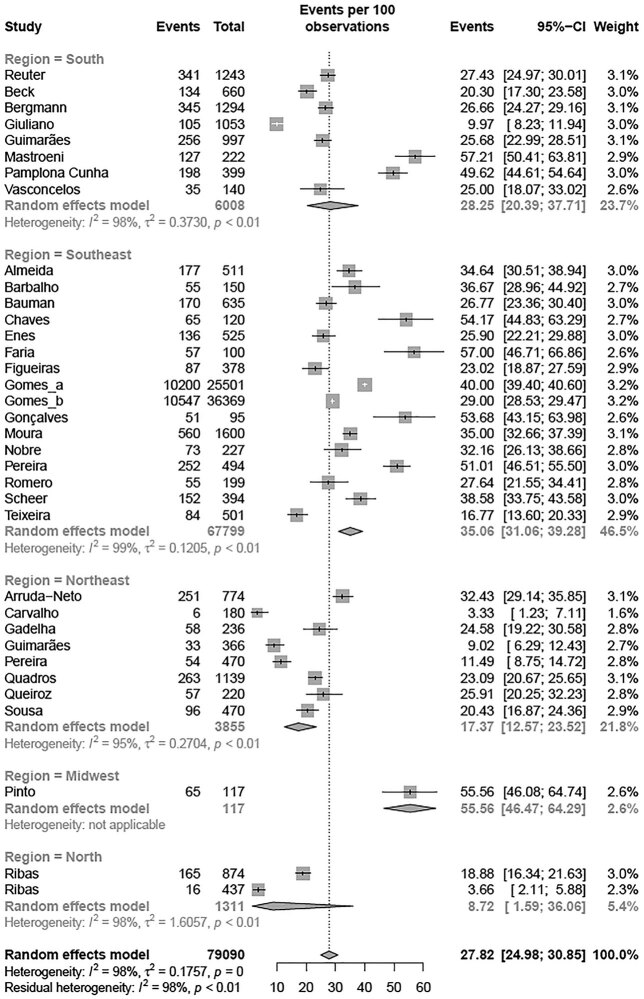
Prevalence of TC according BSC for region of Brazil.

Note: p-value < 0.001.

**Figure S2B:**
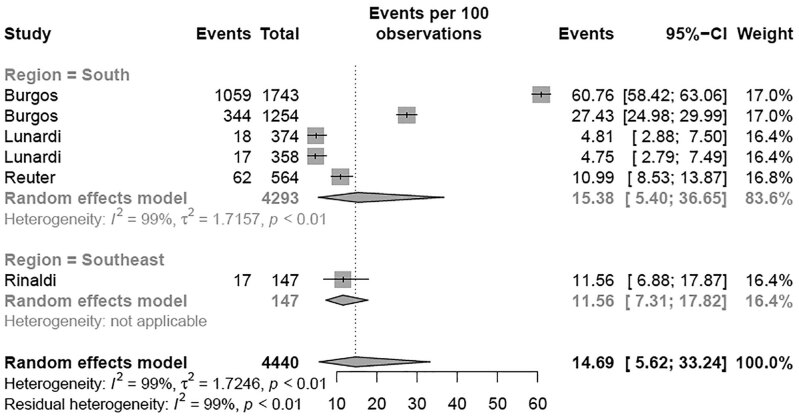
Prevalence of TC according NHLBI for region of Brazil.

Note: p-value = 0.609.

**Figure S3A:**
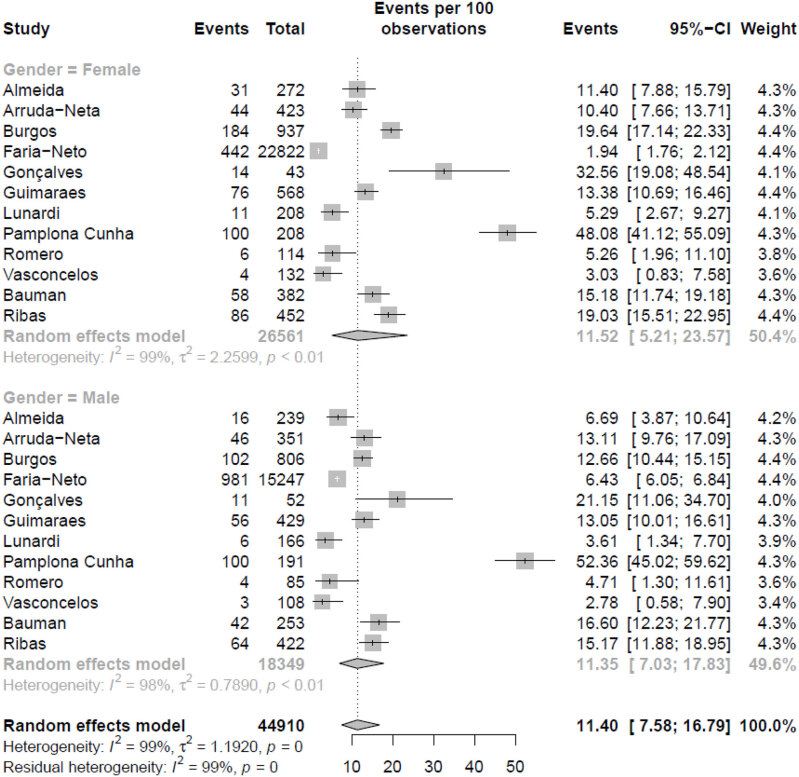
Prevalence of LDL for sex according NHLBI.

**Figure S3B:**
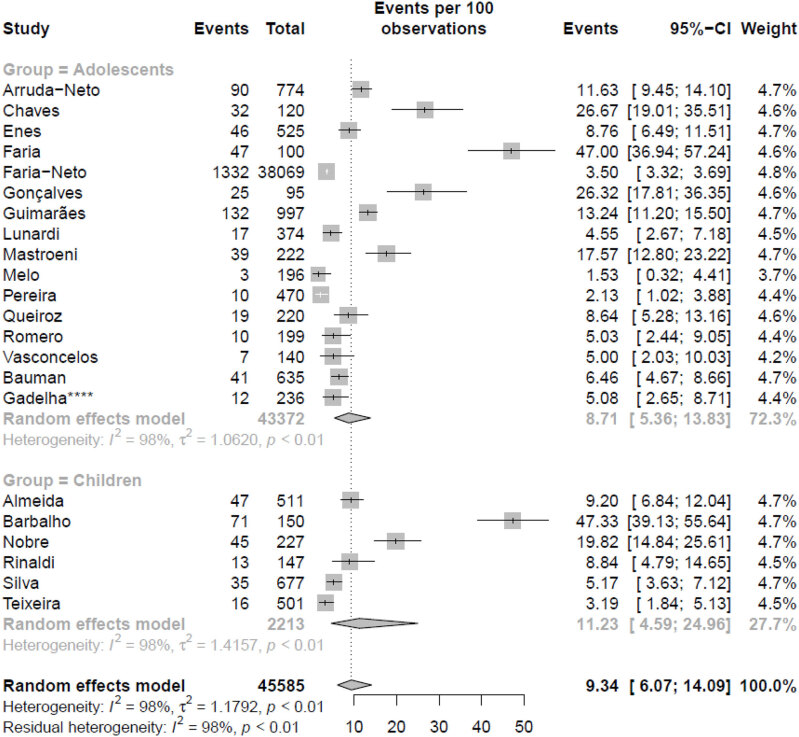
Prevalence of LDL for age groups according NHLBI.

Note: p-value = 0.613.

**Figure S3C:**
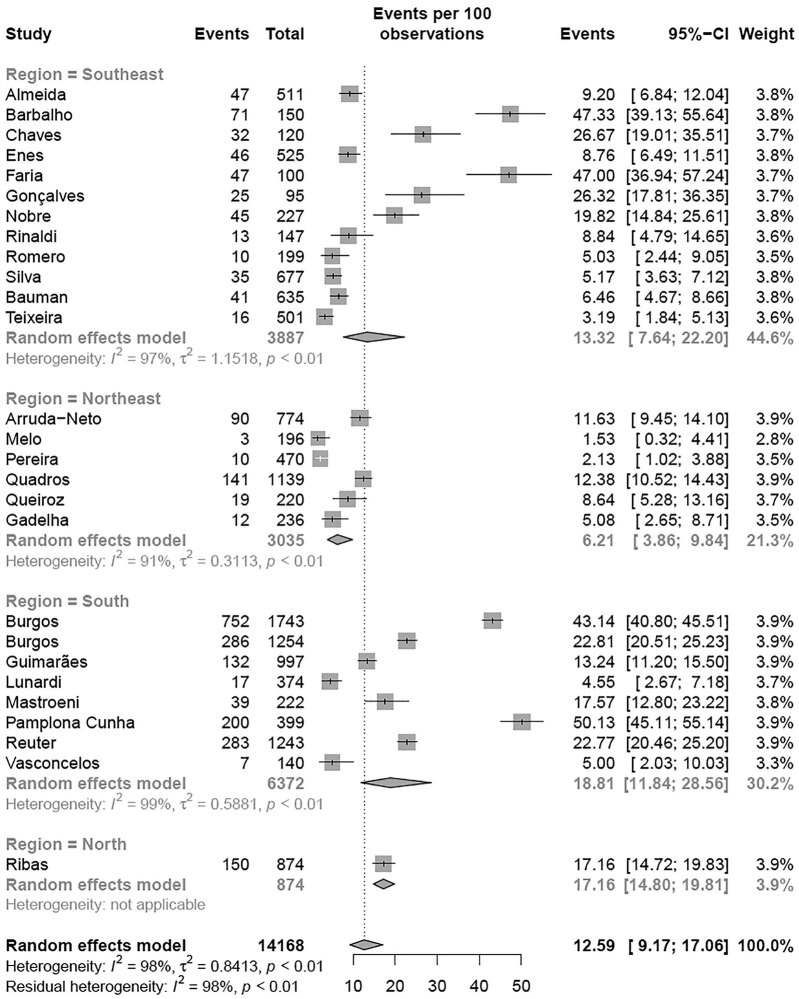
Prevalence of high LDL according NHLBI for region of Brazil

**Figure S3D:**
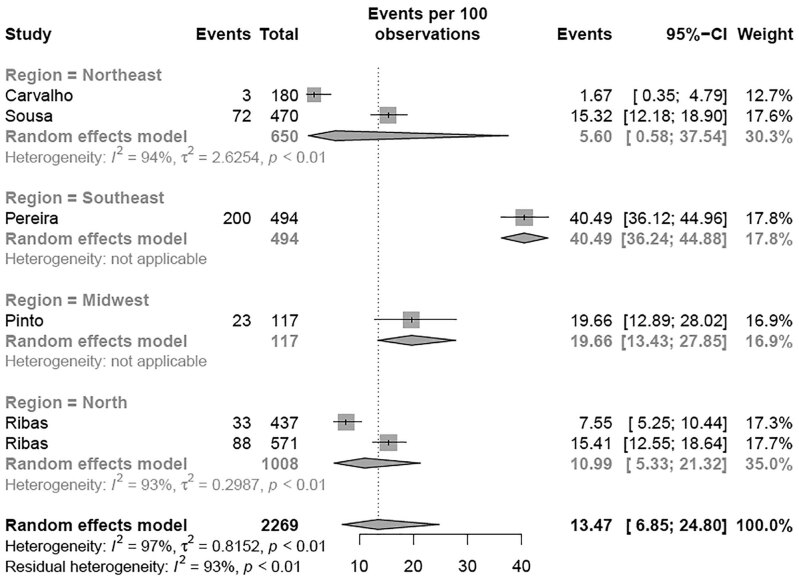
Prevalence of high LDL according BSC for region of Brazil.

**Figure S4A:**
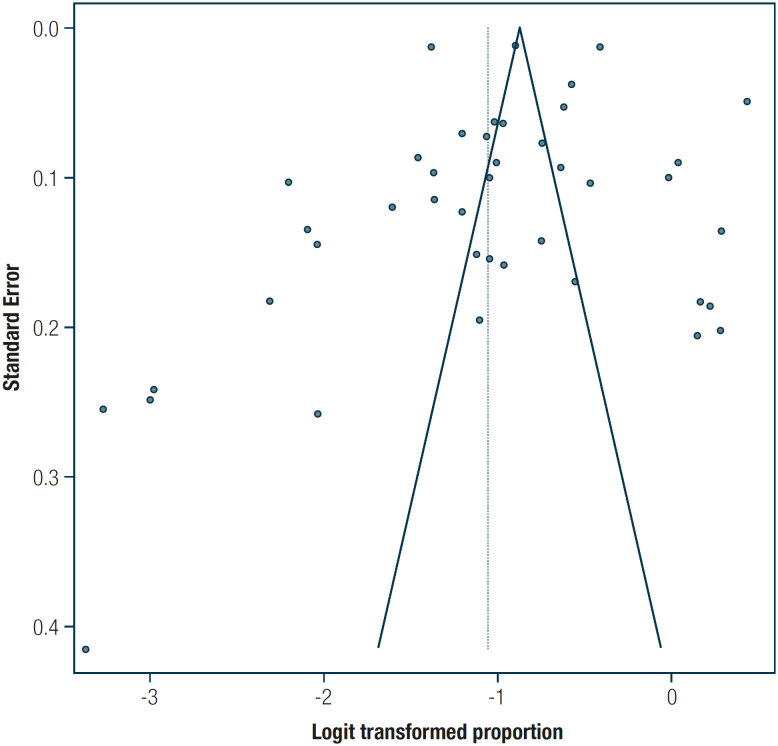
Funnel plot for TC.

**Figure S4B:**
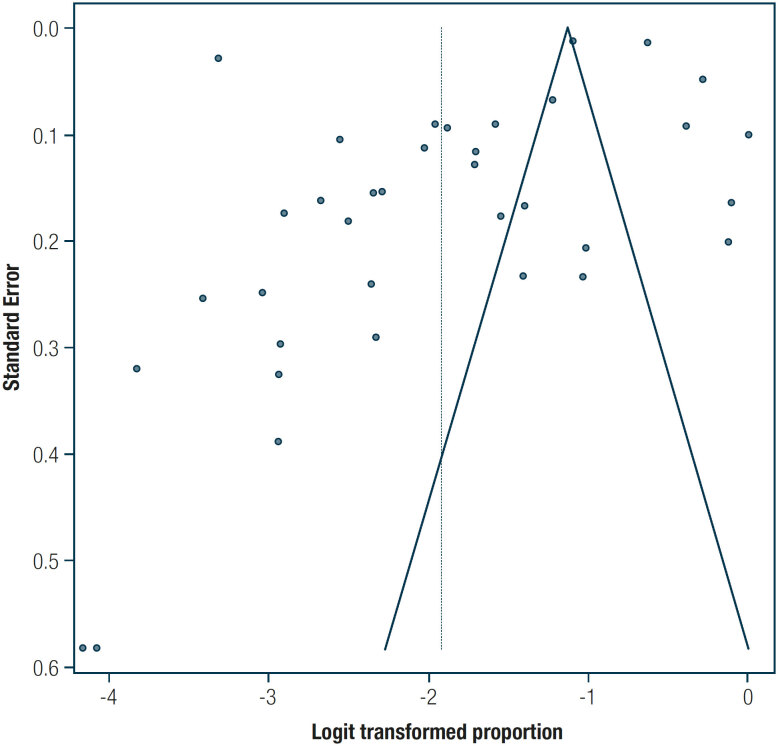
Funnel plot for LDL.

**Figure S4C:**
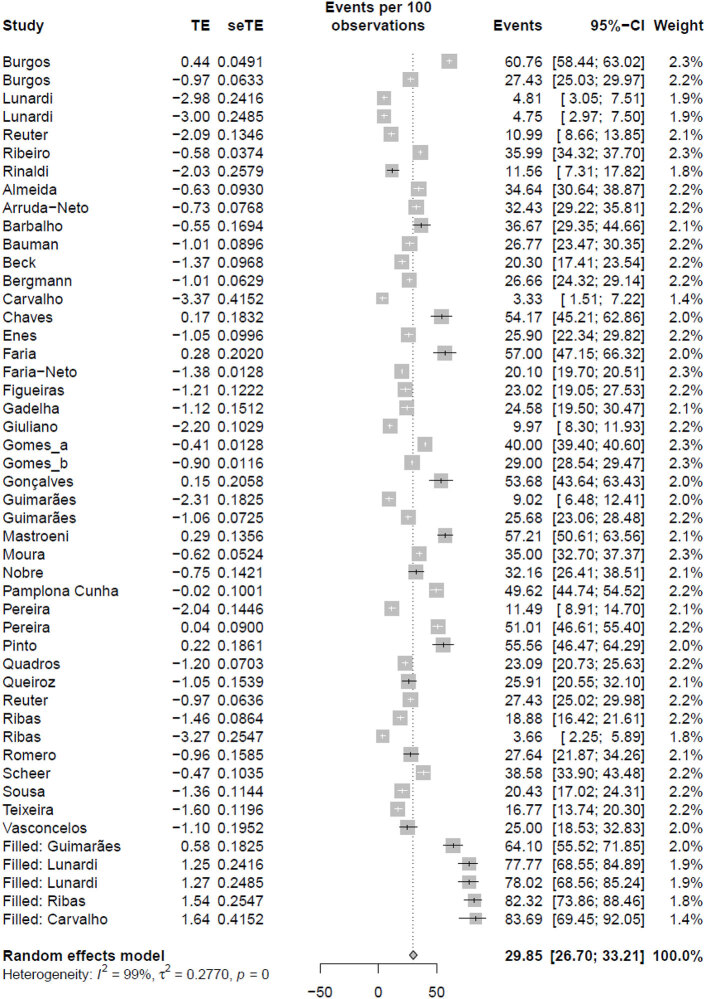
Forest plot with the trim-to-fill correction for TC.

**Figure S4D:**
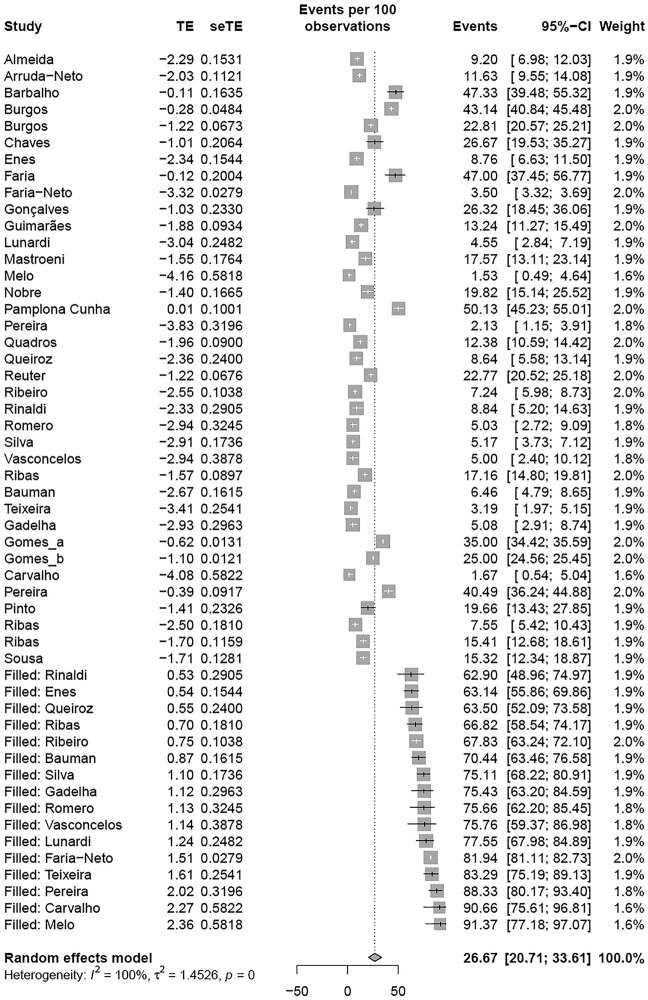
Forest plot with the trim-to-fill correction for LDL.

**Figure S4E:**
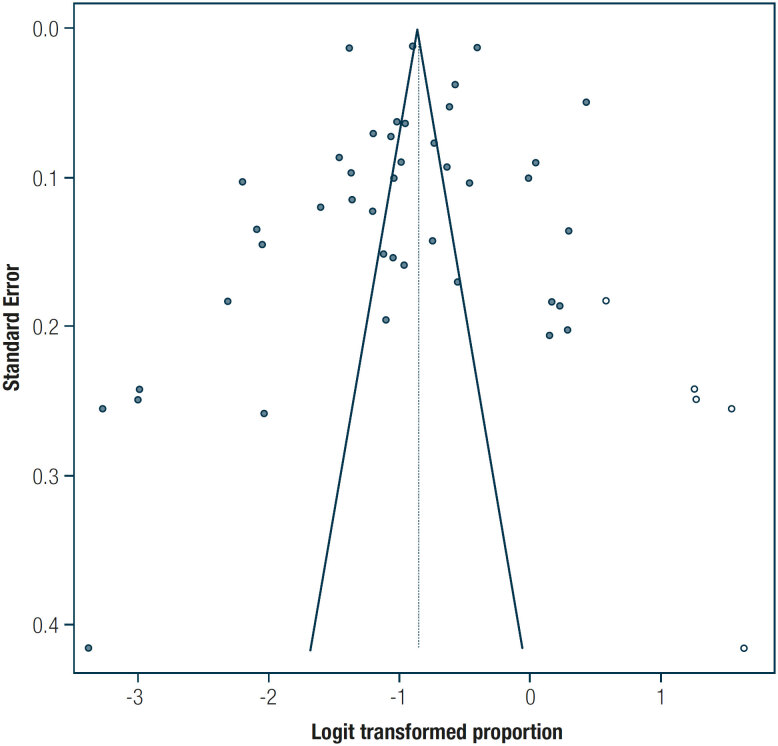
Funnel plot with the trim-to-fill correction for TC.

**Figure S4F:**
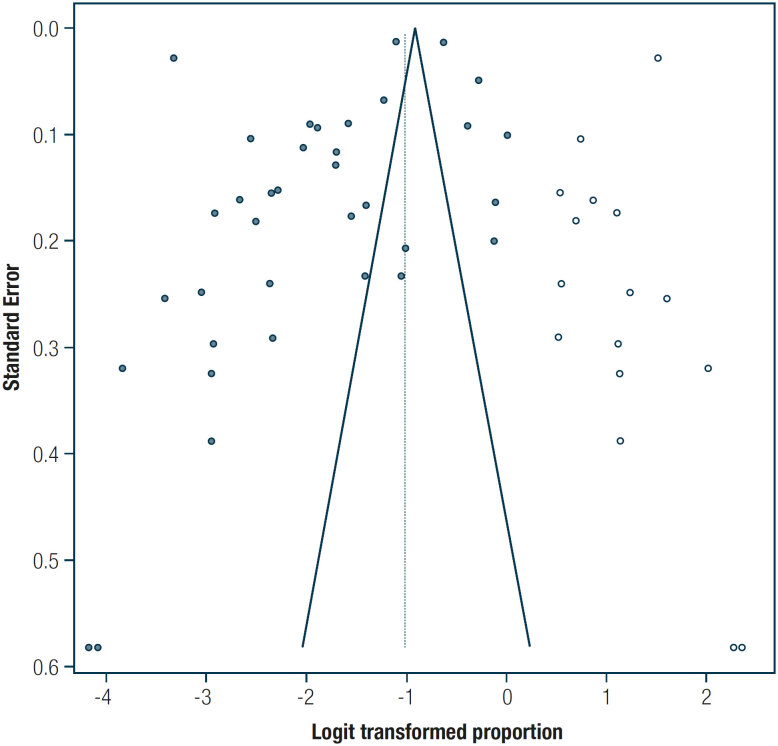
Funnel plot with the trim-to-fill correction for LDL.

## Appendix

**Table S1:** Search strategies each database

Database	Search strategy
PubMed	(((((“Brazil”[Mesh] OR “Brazil”))) AND ((“Child”[Mesh] OR “Child” OR “Children” OR “Adolescent”[Mesh] OR “Adolescent” OR “Adolescents” OR “Adolescence” OR “Teens” OR “Teen” OR “Teenagers” OR “Teenager” OR “Youth” OR “Youths” OR “Adolescents, Female” OR “Adolescent, Female” OR “Female Adolescent” OR “Female Adolescents” OR “Adolescents, Male” OR “Adolescent, Male” OR “Male Adolescent” OR “Male Adolescents”))) AND ((“Dyslipidemias/epidemiology”[Mesh] OR “Dyslipidemias/epidemiology” OR “Dyslipidemias/statistics and numerical data”[Mesh] OR “Dyslipidemias/statistics and numerical data” OR “Dyslipidemia” OR “Dyslipoproteinemias” OR “Dyslipoproteinemia” OR “lipid profile” OR “Cholesterol, VLDL”[Mesh] OR “Cholesterol, VLDL” OR “VLDL Cholesterol” OR “Pre-beta-Lipoprotein Cholesterol” OR “Cholesterol, Pre-beta-Lipoprotein” OR “Pre beta Lipoprotein Cholesterol” OR “Very Low Density Lipoprotein Cholesterol” OR “Prebetalipoprotein Cholesterol” OR “Cholesterol, Prebetalipoprotein” OR “Cholesterol, LDL”[Mesh] OR “Cholesterol, LDL” OR “Low Density Lipoprotein Cholesterol” OR “beta-Lipoprotein Cholesterol” OR “Cholesterol, beta-Lipoprotein” OR “beta Lipoprotein Cholesterol” OR “LDL Cholesterol” OR “Cholesteryl Linoleate, LDL” OR “LDL Cholesteryl Linoleate” OR “Cholesterol, HDL”[Mesh] OR “Cholesterol, HDL” OR “alpha-Lipoprotein Cholesterol” OR “Cholesterol, alpha-Lipoprotein” OR “alpha Lipoprotein Cholesterol” OR “HDL Cholesterol” OR “High Density Lipoprotein Cholesterol” OR “Cholesterol, HDL2” OR “HDL2 Cholesterol” OR “HDL(2) Cholesterol” OR “Cholesterol, HDL3” OR “HDL3 Cholesterol” OR “HDL(3) Cholesterol”))) AND ((“Prevalence”[Mesh] OR “Prevalence” OR “Prevalences”))
SciELO	(“Brazil”) AND (“Child” OR “Children” OR “Adolescent” OR “Adolescents” OR “Adolescence” OR “Teens” OR “Teen” OR “Teenagers” OR “Teenager” OR “Youth” OR “Youths”) AND (“Dyslipidemias” OR “Dyslipidemia” OR “Dyslipoproteinemias” OR “Dyslipoproteinemia” OR “lipid profile” OR “Cholesterol”) AND (“Prevalence” OR “Prevalences”)
Lilacs	tw:((tw:(dislipidemias OR dyslipidemias OR dislipidemias OR colesterol OR cholesterol OR colesterol)) AND (tw:(brasil OR brazil OR brasil)) AND (tw:(criança OR child OR niño OR adolescente OR adolescent OR adolescente)) AND (tw:(prevalência OR prevalence OR prevalencia))) AND (instance:”regional”)
Embase	(‘prevalence’/syn) AND(‘brazil’/syn OR ‘brazil’) AND (‘child’/syn OR ‘adolescent’/syn) AND (‘dyslipidemia’/syn)

## References

[1] Zhang Y, Pletcher MJ, Vittinghoff E, Clemons AM, Jacobs DR, Allen NB (2021). Association Between Cumulative Low-Density Lipoprotein Cholesterol Exposure During Young Adulthood and Middle Age and Risk of Cardiovascular Events. JAMA Cardiol.

[2] Expert Panel on Integrated Guidelines for Cardiovascular Health and Risk Reduction in Children and Adolescents; National Heart, Lung, and Blood Institute (2011). Expert panel on integrated guidelines for cardiovascular health and risk reduction in children and adolescents: summary report. Pediatrics.

[3] Xavier HT, Izar MC, Faria JR, Assad MH, Rocha VZ, Sposito AC (2013). V Diretriz Brasileira de Dislipidemias e Prevenção da Aterosclerose. Arq Bras Cardiol.

[4] Santos RD, Gagliardi ACM, Xavier HT, Magnoni CD, Cassani R, Lottenberg AMP (2013). Sociedade Brasileira de Cardiologia. I Diretriz sobre o consumo de gorduras e saúde cardiovascular. Arq Bras Cardiol.

[5] Sociedade Brasileira de Cardiologia (2001). III Diretrizes Brasileiras Sobre Dislipidemias e Diretriz de Prevenção da Aterosclerose do Departamento de Aterosclerose da Sociedade Brasileira de Cardiologia. Arq Bras Cardiol.

[6] Jokinen E (2015). Obesity and cardiovascular disease. Minerva Pediatr.

[7] Ference BA, Graham I, Tokgozoglu L, Catapano AL (2018). Impact of Lipids on Cardiovascular Health: JACC Health Promotion Series. J Am Coll Cardiol.

[8] Berenson GS (2001). Bogalusa Heart Study: a long-term community study of a rural biracial (black/white) population. Am J Med Sci.

[9] Berenson GS, Wattigney WA, Tracy RE, Ill WPN, Srinivasan SR, Webber LS (1992). Atherosclerosis of the Aorta and Coronary Arteries and Cardiovascular Risk Factors in Persons Aged 6 to 30 Years and Studied at Necropsy (The Bogalusa Heart Study). Am J Cardiol.

[10] Yoon JM (2014). Dyslipidemia in children and adolescents: when and how to diagnose and treat?. Pediatr Gastroenterol Hepatol Nutr.

[11] Tolfrey K (2002). Intraindividual Variability of Children’s Blood Lipid and Lipoprotein Concentrations: A Review. Prev Cardiol.

[12] Fagherazzi S, Dias RL, Bortolon F (2008). Impact of isolated and combined with diet physical exercise on the HDL, LDL, total cholesterol and triglycerides plasma levels. Rev Bras Med Esporte.

[13] Santos MG dos, Pegoraro M, Sandrini F, Macuco EC (2008). Review Article Risk Factors for the Development of Atherosclerosis in Childhood and Adolescence. Arq Bras Cardiol.

[14] Faria JR, Bento VFR, Baena CP, Olandoski M, Gonçalves LGO, Abreu GA (2016). ERICA: prevalência de dislipidemia em adolescentes brasileiros. Rev Saude Publica.

[15] Carlos I, Giuliano B, Caramelli B (2015). Dyslipidemia in childhood and adolescence. Pediatr (São Paulo).

[16] Franca E, Alves JGB (2005). Original Article Dyslipidemia Among Adolescents and Children from Pernambuco. Arq Bras Cardiol.

[17] Grillo LP, Crispim SP, Siebert AN, Andrade ATW, Rossi A, Campos IC (2005). Lipid profile and obesity in low income school children. Rev Bras Epidemiol.

[18] Scherr C, Magalhães CK, Malheiros W (2006). Lipid Profile Analysis in School Children. Arq Bras Cardiol.

[19] Moher D, Liberati A, Tetzlaff J, Altman DG, The PRISMA Group (2009). Preferred Reporting Items for Systematic Reviews and Meta-Analyses: The PRISMA Statement. PLoS Med.

[20] Modesti PA, Reboldi G, Cappuccio FP, Agyemang C, Remuzzi G, Rapi S (2016). Panethnic Differences in Blood Pressure in Europe: A Systematic Review and Meta-Analysis. PLoS One.

[21] Almeida PCD, Silva JP, Pinasco GC, Hegner CC, Mattos DC, Potratz MO (2016). Perfil lipídico em escolares de Vitória – Brasil. J Hum Growth Dev.

[22] Barbalho SM, Oshiiwa M, Lia CSF, Finalli EFR, Paiva ME (2017). Diabetes & Metabolic Syndrome: Clinical Research & Reviews Metabolic syndrome and atherogenic indices in school children: A worrying panorama in Brazil. Diabetes Metab Syndr Clin Res Rev.

[23] Filgueiras MDS, Suhett LG, Silva MA, Rocha NP, Novaes JF (2018). Lower vitamin D intake is associated with low HDL cholesterol and vitamin D insufficiency/deficiency in Brazilian children. Public Health Nutr.

[24] Nobre LN, Lamounier JA, Franceschini SCC (2013). Sociodemographic, anthropometric and dietary determinants of dyslipidemia in preschoolers. J Pediatr (Rio J).

[25] Rinaldi AEM, Oliveira EP de, Moreto F, Gabriel GFCP, Corrente JE, Burini RC (2012). Dietary intake and blood lipid profile in overweight and obese schoolchildren. BMC Res Notes.

[26] Silva NP da, Souza FIS de, Pendezza AI, Fonseca FLA, Hix S, Oliveira AC (2013). Homocysteine and cysteine levels in prepubertal children : Association with waist circumference and lipid profile. Nutrition.

[27] Teixeira FDC, Pereira FEF, Pereira AF, Ribeiro BG (2020). Overweight or obesity and abdominal obesity and their association with cardiometabolic risk factors in Brazilian schoolchildren: A cross-sectional study. Nutrition.

[28] Arruda-Neta ACP, Martins PR, Ferreira FELL (2017). Conicity index as a predictor of changes in the lipid profile of adolescents in a city in Northeast Brazil. Cad Saúde Pública.

[29] Bauman CD, Bauman JM, Mourão DM, Pinho L, Brito MFSF, Carneiro ALG (2020). Dyslipidemia prevalence in adolescents in public schools. Rev Bras Enferm.

[30] Beck CC, Lopes AS, Giuliano ICB, Borgatto AF (2011). Cardiovacular risk factors in adolescents from a town in the Brazilian South: prevalence and association with sociodemographic variables. Rev Bras Epidemiol.

[31] Carvalho DF, Paiva AA, Melo ASO, Ramos AT, Medeiros JS, Medeiros CCM (2007). Blood lipid levels and nutritional status of adolescents. Rev Bras Epidemiol.

[32] Chaves OC, Franceschini SCC, Ribeiro SMR, Sant’Ana LFR, Faria CG, Priore SE (2012). Comparison of the biochemical, anthropometric and body composition variables between adolescents from 10 to 13 years old and their parents. Nutr Hosp.

[33] Enes CC, Silva JR (2018). Association between excess weight and serum lipid alterations in adolescents. Ciênc Saúde Coletiva.

[34] Faria ER, Gontijo CA, Franceschini SCC, Peluzio MCG, Priore SE (2014). Body composition and risk for metabolic alterations in female adolescents. Rev Paul Pediatr.

[35] Gadelha PCFP, de Arruda IKG, Coelho PBP, Queiroz PMA, Maio R, da Silva Diniz A (2019). Consumption of ultraprocessed foods, nutritional status, and dyslipidemia in schoolchildren: a cohort study. Eur J Clin Nutr.

[36] Gonçalves VSS, Chaves OC, Ribeiro SMR, Sant’Ana LFR, Franceschini SCC, Priore SE (2012). Household availability of lipids for consumption and its relationship with serum lipids in adolescents. Rev Paul Pediatr.

[37] Guimarães RF, Silva MP, Mazzardo O, Martins RV, Watanabe PI, Campos W (2019). Metabolic risk factors clustering among adolescents: a comparison between sex, age and socioeconomic status. Ciênc Saúde Coletiva.

[38] Lunardi CC, Petroski ÉL (2008). Índice de Massa Corporal, Circunferência da Cintura e Dobra Cutânea Triciptal na Predição de Alterações Lipídicas em Crianças com 11 Anos de Idade. Arq Bras Cir Dig.

[39] Lunardi CC, Moreira CM, Santos DL dos (2010). Blood Lipids Abnormalities and Overweight Prevalence in Students of Santa Maria, RS, Brazil. Rev Bras Med Esporte.

[40] Mastroeni SSB, Mastroeni MF, Gonçalves MC, Debortoli G, Silva NN, Bernal RTI (2016). Cardiometabolic Risk Markers of Normal Weight and Excess Body Weight in Brazilian Adolescents. Appl Physiol Nutr Metab.

[41] Melo EMFS, Azevedo GD, Silva JD, Lemos TMAM, Maranhão TMO, Freitas AKMSO (2016). Clustering of risk factors for cardiometabolic diseases in low-income, female adolescents. Arch Endocrinol Metab.

[42] Pereira PB, Arruda IKG, Cavalcanti AMTS, Diniz AS (2010). Lipid Profile of Schoolchildren from Recife, PE. Arq Bras Cardiol.

[43] Pinto KAC, Priore SE, Carvalho KMB (2011). Parâmetros metabólicos e fatores de risco associados à obesidade abdominal em adolescentes do sexo feminino de escolas públicas do Distrito Federal (Brasil). Arch Latinoam Nutr.

[44] Queiroz DJM, Silva AS, Dinis AS, Carvalho AT, Araújo EPS, Neves JPR (2019). Vitamin D insufficiency/deficiency and its association with cardiometabolic risk factors in Brazilian adolescents. Nutr Hosp.

[45] Romero A, Rezende LFM, Romero SCS, Villar BS (2014). Relationship between obesity and biochemical markers in Brazilian adolescents. Rev Bras Cineantropom Desempenho Hum.

[46] Scheer C, Helal L, Ferrari F, Belém LJ, Fabiano LCC, Pinheiro LT (2019). The Olympic Experimental Gymnasium Program and its Association with the Prevalence of Cardiovascular Risk Factors in Adolescents: A Cross-Sectional Study. Arq Bras Cardiol.

[47] Sousa MACA, Guimarães ICB, Daltro C, Guimarães AC (2013). Association between Birth Weight and Cardiovascular Risk Factors in Adolescents. Arq Bras Cardiol.

[48] Vasconcelos IQA, Stabelini A, Mascarenhas LPG, Bozza R, Ulbrich AZ, Campos W (2008). Cardiovascular Risk Factors in Adolescents with Different Levels of Energy Expenditure. Arq Bras Cardiol.

[49] Alcântara OD, Silva RCR, Assis AMO, Pinto EJ (2012). Factors associated with dyslipidemia in children and adolescents enrolled in public schools of Salvador, Bahia. Rev Bras Epidemiol.

[50] Bergmann MLA, Bergmann GG, Halpern R, Rech RR, Constanzi CB, Alli LR (2011). Associated Factors to Total Cholesterol: School Based Study in Southern Brazil. Arq Bras Cardiol.

[51] Burgos MS, Tornquist D, Tornquist L, Reuter CP, Garcia EL, Renner JDP (2019). Cardiometabolic risk factors associated with active commuting to school. Rev Paul Pediatr.

[52] Burgos MS, Reuter CP, Possuelo LG, Valim ARM, Renner JDP, Tornquist L (2015). Obesity parameters as predictors of early development of cardiometabolic risk factors. Ciênc Saúde Coletiva.

[53] Guimarães ICB, Guimarães AC (2005). Prevalence of Cardiovascular Risk Factors in Selected Samples of Schoolchildren – Socioeconomic Influence. Prev Cardiol.

[54] Giuliano ICB, Coutinho MSSA, Freitas SFT, Pires MMS, Zunino JN, Ribeiro RQC (2005). Serum Lipids in School Kids and Adolescents from Florianópolis, SC, Brazil – Healthy Floripa 2040 Study. Arq Bras Cardiol.

[55] Gomes ÉIL, Zago VHS, Faria EC (2020). Evaluation of Lipid Profiles of Children and Youth from Basic Health Units in Campinas, SP, Brazil: A Cross-Sectional Laboratory Study. Arq Bras Cardiol.

[56] Moura EC, Castro CM, Mellin AS, Figueiredo DB (2000). Lipidic profile among schoolchildren, Brazil. Rev Saude Publica.

[57] Cunha HP (2014). Avaliação dos fatores de riscos cardiometabólicos e do efeito da atividade física e orientação nutricional em crianças e adolescentes.

[58] Pereira A, Guedes AD, Verreschi ITN, Santos RD, Martinez TLR (2004). Obesity and Its Association with Other Cardiovascular Risk Factors in School Children in Itapetininga, Brazil. Arq Bras Cardiol.

[59] Quadros TMB de, Gordia AP, Silva LR, Silva DAS, Mota J (2016). Epidemiological survey in schoolchildren: determinants and prevalence of cardiovascular risk factors. Cad Saúde Pública.

[60] Quadros TMB de, Gordia AP, Silva RCR da, Silva LR (2015). Predictive capacity of anthropometric indicators for dyslipidemia screening in children and adolescents. J Pediatr (Rio J).

[61] Reuter CP, Burgos L, Camargo MD, Possuelo LG, Reckziegel MB, Reuter ÉM (2013). Prevalence of obesity and cardiovascular risk among children and adolescents in the municipality of Santa Cruz do Sul, Rio Grande do Sul. Sao Paulo Med J.

[62] Reuter CP, Silva PT da, Renner JDP, Mello ED de, Valim AR de M, Pasa L (2016). Dyslipidemia is Associated with Unfit and Overweight-Obese Children and Adolescents. Arq Bras Cardiol.

[63] Ribas SA, Silva LCS (2012). Anthropometric indices predictors of dyslipidemia in children and adolescents from north of Brazil. Nutr Hosp.

[64] Ribas SA, Silva LCS da (2014). Cardiovascular risk and associated factors in schoolchildren in Belém, Pará State, Brazil. Cad Saúde Pública.

[65] Ribas SA, Silva LCS da (2009). Dyslipidemia in Schoolchildren from Private Schools in Belém. Arq Bras Cardiol.

[66] Ribeiro RC, Coutinho M, Bramorski MA, Giuliano IC, Pavan J (2010). Association of the Waist-to-Height Ratio with Cardiovascular Risk Factors in Children and Adolescents: The Three Cities Heart Study. Int J Prev Med.

[67] Sociedade Brasileira de Pediatria (2020). Dislipidemia na criança e no adolescente – Orientações para o pediatra. Departamento Científico de Endocrinologia (2019-2021). Sociedade Brasileira de Pediatria..

[68] Tomeleri CM, Ronque ERV, Silva DRP, Cardoso CG, Fernandes RA, Teixeira DC (2015). Prevalence of dyslipidemia in adolescents: Comparison between definitions. Rev Port Cardiol.

[69] Silveira BM, Kliemann N, Silva DP, Colussi CF, Proença RP (2013). Availability and price of food products with and without trans fatty acids in food stores around elementary schools in low- and medium-income neighborhoods. Ecol Food Nutr.

[70] Restrepo BJ (2020). Intake of trans-fats among US youth declined from 1999-2000 to 2009-2010. Public Health Nutr.

[71] Carmo AS, Assis MM, Cunha CF, Oliveira TRPR, Mendes LL (2018). The food environment of Brazilian public and private schools. Cad Saúde Pública.

[72] Simões BS, Cardoso LO, Benseñor IJM, Schmidt MI, Duncan BB, Luft VC (2018). Consumption of ultra-processed foods and socioeconomic position : a cross-sectional analysis of the Brazilian Longitudinal Study of Adult Health. Cad Saúde Pública.

[73] Instituto Brasileiro de Geografia e Estatística (IBGE) (2011). Pesquisa de orçamentos familiares 2008-2009: análise do consumo alimentar pessoal no Brasil.

[74] Meng H, Zhu L, Kord-Varkaneh H, O Santos H, Tinsley GM, Fu P (2020). Effects of intermittent fasting and energy-restricted diets on lipid profile: A systematic review and meta-analysis. Nutrition.

[75] Song SJ, Lee JE, Paik HY, Park MS, Song YJ (2012). Dietary patterns based on carbohydrate nutrition are associated with the risk for diabetes and dyslipidemia. Nutr Res Pract.

[76] Bahadoran Z, Mirmiran P, Hosseini-Esfahabni F, Sadeghi M, Azizi F (2013). Dietary Protein, Protein to Carbohydrate Ratio and Subsequent Changes in Lipid Profile after a 3-Year Follow-Up: Tehran Lipid and Glucose Study. Iran J Public Health.

[77] Bel-Serrat S, Mouratidou T, Huybrechts I, Cuenca-García M, Manios Y, Gómez-Martínez S (2014). The role of dietary fat on the association between dietary amino acids and serum lipid profile in European adolescents participating in the HELENA Study. Eur J Clin Nutr.

[78] Miller JC, Smith C, Williams SM, Mann JI, Brown RC, Parnell WR (2016). Trends in serum total cholesterol and dietary fat intakes in New Zealand between 1989 and 2009. Aust N Z J Public Health.

[79] Cureau FV, da Silva TL, Bloch KV, Fujimori E, Belfort DR, de Carvalho KM (2016). ERICA: leisure-time physical inactivity in Brazilian adolescents. Rev Saude Publica.

[80] Lazzoli JK, Nóbrega ACL, Carvalho T, Oliveira MAB, Teixeira JAC, Leitão MB (1998). Atividade física e saúde na infância e adolescência. Rev Bras Med Esporte.

[81] Schwarzfischer P, Gruszfeld D, Stolarczyk A, Ferre N, Escribano J, Rousseaux D (2019). Physical Activity and Sedentary Behavior From 6 to 11 Years. Pediatrics.

[82] Labarthe DR, Nichaman MZ, Harrist RB, Grunbaum JA, Dai S (1997). Development of cardiovascular risk factors from ages 8 to 18 in Project HeartBeat! Study design and patterns of change in plasma total cholesterol concentration. Circulation.

[83] Freedman DS, Bowman BA, Srinivasan SR, Berenson GS, Otvos JD (2001). Distribution and correlates of high-density lipoprotein subclasses among children and adolescents. Metabolism.

[84] Srinivasan SR, Myers L, Berenson GS (2002). Distribution and correlates of non-high-density lipoprotein cholesterol in children: the Bogalusa Heart Study. Pediatrics.

[85] Spinneker A, Egert S, González-Gross M, Breidenassel C, Albers U, Stoffel-Wagner B (2012). Lipid, lipoprotein and apolipoprotein profiles in European adolescents and its associations with gender, biological maturity and body fat - the HELENA Study. Eur J Clin Nutr.

[86] Schienkiewitz A, Truthmann J, Ernert A, Wiegand S, Schwab KO, Scheidt-Nave C (2019). Age, maturation and serum lipid parameters: findings from the German Health Survey for Children and Adolescents. BMC Public Health.

[87] Higgins JPT, Thomas J, Chandler J, Cumpston M, Li T, Page MJ (2019). Cochrane Handbook for Systematic Reviews of Interventions version 6.0 (updated July 2019).

